# Supplementary material for: Differentiable simulation to develop molecular dynamics force fields for disordered proteins
Source: Chem Sci. 2024 Feb 21;15(13):4897–909. doi: 10.1039/d3sc05230c (PMC10966991; doi:10.1039/d3sc05230c)
Supplement: SC-015-D3SC05230C-s001 [file SC-015-D3SC05230C-s001.pdf]

Electronic Supplementary Material (ESI) for Chemical Science.
This journal is © The Royal Society of Chemistry 2024

Differentiable simulation to develop molecular dynamics force fields for disordered proteins

Joe G Greener

Supplementary Data

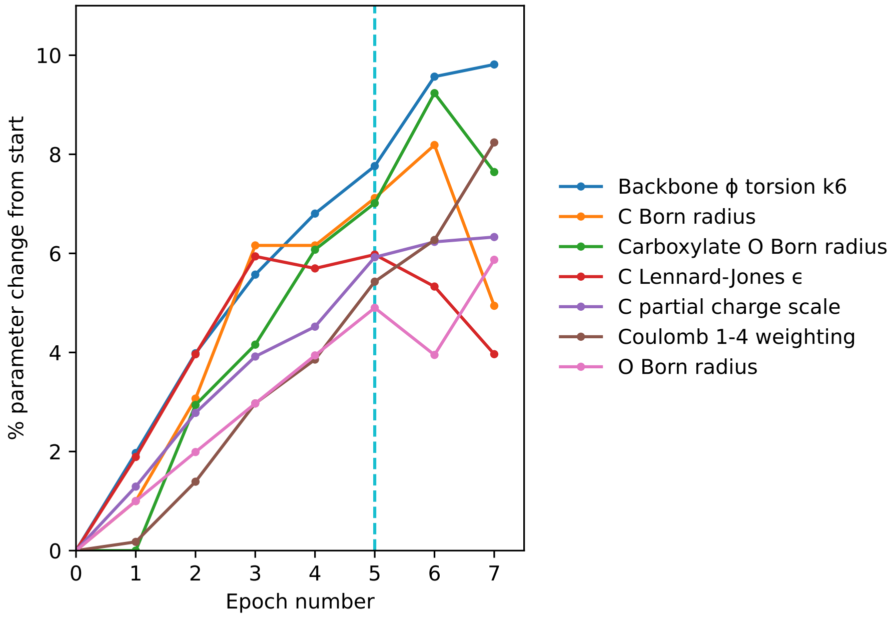

Figure S1 Parameter changes during training. The change in absolute value of 7 parameters that change by at least 4.5% in absolute value from the starting values in a99SB-*disp*+GBNeck2 are shown. Parameters after epoch 5 were used in GB99dms, as indicated by a cyan line.

Table S1 Parameters in GB99dms. The other parameters not changed during training are the same as in a99SB-*disp* or GBNeck2. The atom type HO LJ parameters are zero in a99SB-*disp* so are not modified. This table is available in CSV format in the data repository.

**Parameter**	**Value in GB99dms**	**Value in a99SB-*disp* or GBNeck2**	**Change over training**	**% change over training**
atom CA charge scale	0.996	1.0	-0.00395	-0.39%
atom CA LJ σ / nm	0.342	0.34	0.00183	0.54%
atom CA LJ ε / kJ mol^{-1}	0.364	0.36	0.00452	1.26%
atom CT charge scale	1.01	1.0	0.0104	1.04%
atom CT LJ σ / nm	0.337	0.34	-0.00304	-0.89%
atom CT LJ ε / kJ mol^{-1}	0.447	0.458	-0.0111	-2.43%
atom C charge scale	1.06	1.0	0.0592	5.92%
atom C LJ σ / nm	0.352	0.34	0.0117	3.43%
atom C LJ ε / kJ mol^{-1}	0.381	0.36	0.0215	5.98%
atom C8 charge scale	0.999	1.0	-0.000894	-0.09%
atom C8 LJ σ / nm	0.339	0.34	-0.000537	-0.16%
atom C8 LJ ε / kJ mol^{-1}	0.457	0.458	-0.00115	-0.25%
atom C9 charge scale	1.0	1.0	-7.96e-05	-0.01%
atom C9 LJ σ / nm	0.339	0.34	-0.000495	-0.15%
atom C9 LJ ε / kJ mol^{-1}	0.456	0.458	-0.00204	-0.45%
atom N charge scale	1.0	1.0	0.00188	0.19%
atom N LJ σ / nm	0.339	0.325	0.0135	4.16%
atom N LJ ε / kJ mol^{-1}	0.737	0.711	0.026	3.66%
atom N3 charge scale	0.999	1.0	-0.000968	-0.10%
atom N3 LJ σ / nm	0.325	0.325	0.000179	0.05%
atom N3 LJ ε / kJ mol^{-1}	0.711	0.711	0.000102	0.01%
atom O charge scale	1.01	1.0	0.00632	0.63%
atom O LJ σ / nm	0.296	0.296	0.000153	0.05%
atom O LJ ε / kJ mol^{-1}	0.863	0.879	-0.0159	-1.81%
atom O2 charge scale	1.01	1.0	0.00893	0.89%
atom O2 LJ σ / nm	0.298	0.296	0.00192	0.65%
atom O2 LJ ε / kJ mol^{-1}	0.879	0.879	0.000523	0.06%
atom OH charge scale	1.0	1.0	0.00111	0.11%
atom OH LJ σ / nm	0.305	0.307	-0.00125	-0.41%
atom OH LJ ε / kJ mol^{-1}	0.879	0.88	-0.00152	-0.17%
atom H charge scale	1.0	1.0	0.000143	0.01%
atom H LJ σ / nm	0.105	0.107	-0.00178	-1.66%
atom H LJ ε / kJ mol^{-1}	0.065	0.0657	-0.000736	-1.12%
atom H1 charge scale	1.0	1.0	-9.15e-05	-0.01%
atom H1 LJ σ / nm	0.246	0.247	-0.00136	-0.55%
atom H1 LJ ε / kJ mol^{-1}	0.0656	0.0657	-0.000109	-0.17%
atom HA charge scale	1.0	1.0	-9.91e-06	-0.00%
atom HA LJ σ / nm	0.261	0.26	0.000723	0.28%
atom HA LJ ε / kJ mol^{-1}	0.063	0.0628	0.00022	0.35%
atom HC charge scale	1.0	1.0	-0.000251	-0.03%
atom HC LJ σ / nm	0.259	0.265	-0.00634	-2.39%
atom HC LJ ε / kJ mol^{-1}	0.0637	0.0657	-0.00201	-3.06%
atom HO charge scale	1.0	1.0	-9.49e-05	-0.01%
atom HP charge scale	1.0	1.0	2.04e-05	0.00%
atom HP LJ σ / nm	0.196	0.196	0.000429	0.22%
atom HP LJ ε / kJ mol^{-1}	0.0658	0.0657	8.14e-05	0.12%

**Parameter**	**Value in GB99dms**	**Value in a99SB-*disp* or GBNeck2**	**Change over training**	**% change over training**
Lennard-Jones 1-4 weighting	0.51	0.5	0.00979	1.96%
Coulomb 1-4 weighting	0.879	0.833	0.0452	5.43%
torsion -/C/N/- k1 / kJ mol^{-1}	-10.5	-10.5	0.00207	0.02%
torsion -/CA/CA/- k1 / kJ mol^{-1}	-15.2	-15.2	-7.93e-06	-0.00%
torsion -/CT/C8/- k1 / kJ mol^{-1}	0.648	0.649	-0.00068	-0.10%
torsion -/CT/C9/- k1 / kJ mol^{-1}	0.649	0.649	0.000208	0.03%
torsion -/CT/CT/- k1 / kJ mol^{-1}	0.655	0.651	0.00405	0.62%
torsion -/CT/N3/- k1 / kJ mol^{-1}	0.651	0.651	6.06e-05	0.01%
torsion C/N/CT/C k1 / kJ mol^{-1}	-0.14	-0.142	0.00256	1.80%
torsion C/N/CT/C k2 / kJ mol^{-1}	1.4	1.4	0.000432	0.03%
torsion C/N/CT/C k3 / kJ mol^{-1}	2.27	2.28	-0.00382	-0.17%
torsion C/N/CT/C k4 / kJ mol^{-1}	0.332	0.335	-0.00223	-0.67%
torsion C/N/CT/C k5 / kJ mol^{-1}	1.67	1.67	-0.00378	-0.23%
torsion CT/CT/C/N k1 / kJ mol^{-1}	0.837	0.837	-0.000137	-0.02%
torsion CT/CT/C/N k2 / kJ mol^{-1}	0.837	0.837	0.000467	0.06%
torsion CT/CT/C/N k3 / kJ mol^{-1}	1.67	1.67	-0.00251	-0.15%
torsion CT/CT/N/C k1 / kJ mol^{-1}	8.37	8.37	-0.00109	-0.01%
torsion CT/CT/N/C k2 / kJ mol^{-1}	8.37	8.37	0.00268	0.03%
torsion CT/CT/N/C k3 / kJ mol^{-1}	1.67	1.67	-0.0032	-0.19%
torsion H/N/C/O k1 / kJ mol^{-1}	8.37	8.37	1.84e-05	0.00%
torsion H/N/C/O k2 / kJ mol^{-1}	-10.5	-10.5	-7.33e-05	-0.00%
torsion H1/CT/C/O k1 / kJ mol^{-1}	3.35	3.35	0.000823	0.02%
torsion H1/CT/C/O k2 / kJ mol^{-1}	-0.332	-0.335	0.00321	0.96%
torsion HC/CT/C4/CT k1 / kJ mol^{-1}	0.67	0.669	0.000134	0.02%
torsion N/CT/C/N k1 / kJ mol^{-1}	2.72	2.72	-0.00113	-0.04%
torsion N/CT/C/N k2 / kJ mol^{-1}	-0.825	-0.824	-0.000732	-0.09%
torsion N/CT/C/N k3 / kJ mol^{-1}	6.05	6.05	0.000215	0.00%
torsion N/CT/C/N k4 / kJ mol^{-1}	2.0	2.0	0.000106	0.01%
torsion N/CT/C/N k5 / kJ mol^{-1}	-0.0776	-0.0799	0.00232	2.90%
torsion N/CT/C/N k6 / kJ mol^{-1}	-0.0154	-0.0167	0.0013	7.76%
torsion N/CT/C/N k7 / kJ mol^{-1}	-1.07	-1.07	0.0	0.00%
torsion N/CT/C/N k8 / kJ mol^{-1}	0.314	0.314	0.0	0.00%
torsion N/CT/C/N k9 / kJ mol^{-1}	0.238	0.238	0.0	0.00%
torsion N/CT/C/N k10 / kJ mol^{-1}	0.105	0.105	0.0	0.00%
torsion N/CT/C/N k11 / kJ mol^{-1}	-0.046	-0.046	0.0	0.00%

Parameter	Value in GB99dms	Value in a99SB-*disp* or GBNeck2	Change over training	% change over training
GB atom radius C / nm	0.182	0.17	0.0121	7.12%
GB atom radius N / nm	0.153	0.155	-0.002	-1.29%
GB atom radius O / nm	0.143	0.15	-0.00735	-4.90%
GB atom radius O_CAR / nm	0.13	0.14	-0.00982	-7.01%
GB atom radius H / nm	0.124	0.12	0.00431	3.59%
GB atom radius H_N / nm	0.125	0.13	-0.00504	-3.88%
GB atom parameter α C	0.75	0.734	0.016	2.18%
GB atom parameter β C	0.487	0.506	-0.0197	-3.89%
GB atom parameter γ C	0.214	0.206	0.00859	4.17%
GB atom parameter α N	0.507	0.503	0.00391	0.78%
GB atom parameter β N	0.318	0.317	0.0013	0.41%
GB atom parameter γ N	0.191	0.193	-0.00227	-1.18%
GB atom parameter α O	0.837	0.868	-0.0313	-3.60%
GB atom parameter β O	0.899	0.877	0.0227	2.59%
GB atom parameter γ O	0.389	0.388	0.00134	0.35%
GB atom parameter α H	0.788	0.788	-0.000574	-0.07%
GB atom parameter β H	0.799	0.799	0.000786	0.10%
GB atom parameter γ H	0.436	0.437	-0.00122	-0.28%
GB screening parameter C	1.1	1.06	0.0456	4.30%
GB screening parameter N	0.736	0.734	0.00278	0.38%
GB screening parameter O	1.07	1.06	0.00517	0.49%
GB screening parameter H	1.43	1.43	0.000751	0.05%
GB neck cutoff / nm	0.68	0.68	0.0	0.00%
GB neck scale	0.853	0.827	0.0257	3.11%
GB offset / nm	0.0195	0.0195	-1.38e-05	-0.07%
GB probe radius / nm	0.134	0.14	-0.00574	-4.10%
GB SA factor / kJ mol^{-1} nm^{-2}	28.4	28.4	-0.000219	-0.00%

Table S2 Consistency of gradients. In each case the gradients for the first epoch of training of the run yielding the final model are compared to the gradients for the first epoch of training of the run described. The fraction of corresponding parameter gradients with the same sign and the Pearson correlation coefficient of corresponding parameter gradients are calculated for each protein and the mean over proteins is shown. For the "Noise added to parameters" run, all starting parameter values were multiplied by a number uniformly-distributed in 0.95 to 1.05.

Run details	Fraction same sign	Correlation
Repeat 1	0.775	0.878
Repeat 2	0.820	0.901
0.5 fs time step, 2.5 ns simulations	0.694	0.777
Noise added to parameters	0.767	0.859
